# Natural Variation in Vif: Differential Impact on APOBEC3G/3F and a Potential Role in HIV-1 Diversification

**DOI:** 10.1371/journal.ppat.0010006

**Published:** 2005-07-22

**Authors:** Viviana Simon, Veronique Zennou, Deya Murray, Yaoxing Huang, David D Ho, Paul D Bieniasz

**Affiliations:** Aaron Diamond AIDS Research Center, The Rockefeller University, New York, New York, United States of America; King's College London, United Kingdom

## Abstract

The HIV-1 Vif protein counteracts the antiviral activity exhibited by the host cytidine deaminases APOBEC3G and APOBEC3F. Here, we show that defective *vif* alleles can readily be found in HIV-1 isolates and infected patients. Single residue changes in the Vif protein sequence are sufficient to cause the loss of Vif-induced APOBEC3 neutralization. Interestingly, not all the detected defects lead to a complete inactivation of Vif function since some mutants retained selective neutralizing activity against APOBEC3F but not APOBEC3G or vice versa. Concordantly, independently hypermutated proviruses with distinguishable patterns of G-to-A substitution attributable to cytidine deamination induced by APOBEC3G, APOBEC3F, or both enzymes were present in individuals carrying proviruses with completely or partly defective Vif variants. Natural variation in Vif function may result in selective and partial neutralization of cytidine deaminases and thereby promote viral sequence diversification within HIV-1 infected individuals.

## Synopsis

Host cells express DNA-editing enzymes, termed APOBEC3G and APOBEC3F, that are capable of profoundly attenuating the replication of retroviruses. However, human immunodeficiency viruses use an efficient approach to “neutralize” these cellular enzymes: specifically, the viral gene *vif* counters the antiretroviral activity of APOBEC3G and APOBEC3F by inducing their degradation.

This study systematically analyzes natural variation in the ability of Vif to neutralize APOBEC-mediated HIV-1 editing and demonstrates that some Vif mutants selectively fail to efficiently “silence” APOBEC3 enzymes. These findings extend the conventional view that errors of reverse transcription and recombination are largely responsible for the rapid evolution of HIV-1 in vivo. Indeed, variation in Vif function may profoundly impact the extent and direction of viral sequence evolution within HIV-1-infected individuals. Thus, innate cellular defenses that likely evolved to restrict retroviral replication might have been usurped by HIV to accelerate viral sequence diversification and escape from immune control and inhibition by antiretroviral drugs.

## Introduction

A variety of intrinsic mechanisms that protect organisms from retroviral infection and corresponding viral escape strategies have evolved as a consequence of the coexistence of retroviruses and vertebrates [1–4]. Lentiviruses, e.g., express Vif proteins that counter the antiretroviral activities of DNA-editing enzymes APOBEC3G [5–8] and APOBEC3F [9–12] by inducing their degradation by proteasomes [7,8,13–15].

While both APOBEC3G and APOBEC3F (and, to a lesser extent, APOBEC3B) exhibit anti-HIV-1 activity [9–12,16], APOBEC3F appears less potent than APOBEC3G and is partially resistant to HIV-1 Vif [11]. Importantly, the expression of APOBEC3G and APOBEC3F seems to be coordinated, probably because they have arisen by gene duplication/ recombination and possess highly homologous promoters. Both are expressed in cell populations susceptible to HIV-1 infection [9,11,17].

APOBEC3G- or APOBEC3F-catalyzed deamination is not highly sequence-specific, and many cytidines on the minus strand of nascent retroviral genomes or reporter genes can be deaminated [12,14,18–20]. However, dinucleotide context strongly influences the efficiency with which cytidine deamination occurs, such that 5′-dCdC and 5′-dTdC are the favored dinucleotides targeted by APOBEC3G and APOBEC3F, respectively [9,11,20,21]. Cytidine deamination events can be manifested as G-to-A changes on the plus strand of the viral genome [11,12,14,19–21], and incomplete suppression of cytidine deaminases by HIV-1 Vif might explain the adenosine biased nucleotide composition of the HIV-1 genome. Similarly, the occasional occurrence of hypermutated genomes containing numerous G-to-A changes [22–26] could reflect occasional inactivating mutations in HIV-1 Vif. Overall, it seems likely that Vif-mediated protection of HIV-1 genomes from host-mediated viral cDNA deamination in vivo is not absolute.

Thus far, analyses of natural variation in HIV-1 Vif function have been restricted to prediction of defects resulting from gross mutations (e.g., missing translation initiation or premature stop codons). Indeed, some reports suggest that long-term non-progressors (LTNPs) harbor grossly mutated Vif more frequently than do those with progressive HIV disease [27]. However, this is not a consistent finding [28,29]. Because natural variation in the function of apparently intact HIV-1 *vif* alleles remains largely unexplored, we examined the extent of variation in APOBEC3G and APOBEC3F neutralizing activity among naturally occurring HIV-1 Vif variants. Surprisingly, we found that apparently intact but inactive Vif variants are frequently detected in viral sequences from very different sources. Importantly, we also found Vif variants that selectively fail to neutralize APOBEC3G and/or APOBEC3F activity, as well as proviruses in LTNPs that appeared to be independently hypermutated as a result of APOBEC3G or APOBEC3F action. These studies indicate that sporadic inactivation of Vif likely occurs rather frequently in vivo, and that natural variation in Vif function is likely to profoundly impact the extent and direction of viral sequence evolution within HIV-1-infected individuals.

## Results

### Naturally Occurring Vif Diversity

Two distinct sources of Vif alleles were selected in order to either maximize or minimize the occurrence of inactive variants. The first group was derived from uncultured DNA isolated from peripheral blood mononuclear cells (PBMCs) of three extensively studied individuals (P1, P2, and P3) with non-progressive HIV-1 infection for more than 20 y [28, 30–35]. We reasoned that the very low (undetectable) level of HIV-1 replication that occurs in these individuals would maximize the chances of detecting naturally occurring defective *vif* alleles, because replication-defective HIV-1 variants should not be obscured by superimposed replicating virus. Moreover, some viral DNA sequences in these individuals contained numerous G-to-A changes in the 5′LTR (P2) [33] and in *gag* (P3) [31]. Amplification and sequence analysis of APOBEC3G mRNA from all three LTNPs excluded the possibility that hypermutation of viral sequences was due to the existence of a naturally occurring Vif-resistant APOBEC3G mutant (data not shown).

A second set of Vif variants was derived from four short-term HIV-1 isolates (V1, V2, V3, and V4) [36]. Vif variants were cloned after a limited period of co-culture of patient cells with “non-permissive” PBMCs, so as to provide a selective pressure for active *vif* alleles while minimizing the introduction of new mutations. V1, V3, and V4 were obtained from recently infected individuals, and V2 was obtained from a chronically infected patient. Table S1 provides a summary of characteristics of the patients from which viral isolates and DNA were obtained.

**Figure 1:**
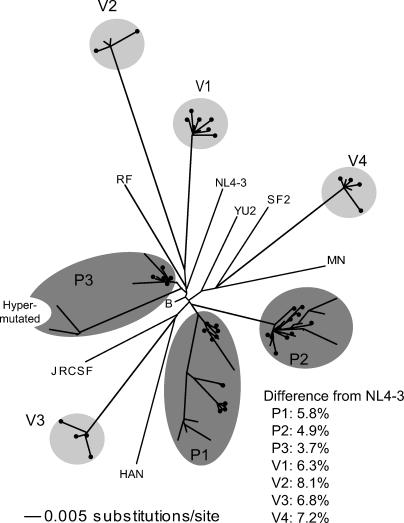
Naturally Occurring *vif* Sequence Variation The phylogenetic relationships among 79 independent *vif* sequences derived from patients (P1, P2, P3) and viral isolates (V1, V2, V3, V4) were analyzed using the Neighbor-joining method. Seven subtype B reference sequences and a consensus subtype B sequence were also included. A cluster of hypermutated *vif* sequences found in P3 is indicated. The 40 distinct protein variants selected for functional testing are identified by •. For each patient and isolate, an individual *vif* consensus sequence was generated and the percent divergence of each *vif* consensus sequence from the NL4–3 *vif* referenced is shown.

The phylogenetic relationships among a total of 79 independent *vif* sequences obtained from these sources is depicted in Figure 1. For each of the LTNPs, a set of Vif sequences collected in 1993–1994 [28] as well as from one or two additional time-points were included (see Material and Methods). Because the neighbor-joining analysis revealed no discernable temporal influence on phylogeny in P1, P2, and P3, the observed intra-individual sequence variation likely represents a spectrum of archived proviruses rather than ongoing sequence evolution. Consensus *vif* sequences from each individual or isolate diverged by 3–9% from a prototype *vif* allele (from HIV-1 NL4–3), a value typical of North American subtype-B *vif* sequences (Figure 1).

While the majority of *vif* sequences derived from LTNPs and viral isolates encoded a full-length protein (192 residues), six sequences (from P2, P3, and V4) contained one or more premature stop codons. Interestingly, each of these resulted from a G-to-A mutation in the context of a Trp codon (TGG to TAG). In addition, a subpopulation of obviously defective Vif sequences from P3 (Figure 1) contained numerous G-to-A substitutions. Thus, expression of intact Vif proteins can be abolished by relatively rare G-to-A substitutions at tryptophan sites, or by overt hypermutation.

### Frequent Occurrence of Non-Functional Vif

**Figure 2:**
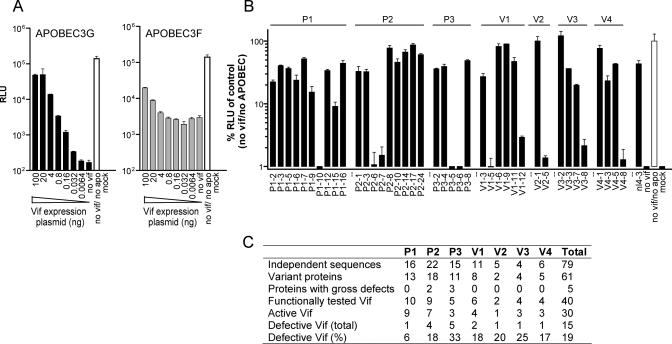
Activity of Vif Variants from Patients and Viral Isolates (A) Infectivity of HIV-1 vector particles generated by transient transfection of 293T cells in the presence or absence of fixed amounts of APOBEC3G or APOBEC3F and the indicated amounts of HIV-1 NL4–3 Vif expression plasmids was determined, as described in the Material and Methods. Representative results from one out of three independent experiments are depicted. Infectivity measurements were performed in duplicate assays, and the error bars represent the standard deviation of the RLU values. RLU, relative light units. (B) APOBEC3G neutralization by Vif proteins from LTNPs (P1, P2, P3) and viral isolates (V1, V2, V3, V4). The infectivity of particles generated in the presence of APOBEC3G and each Vif protein is expressed relative to the infectivity of particles generated in the absence of APOBEC3G and Vif. Each Vif protein is identified by its source (e.g., P1, V1) and a variant number (e.g., P1–2). The data represents the average infectivity values of at least three independent experiments, with the error bars showing the standard deviations. (C) Summary of the properties of Vif variants. Independent sequences were defined as alleles that were derived from different PCR reactions or had different nucleotide sequences. Because some changes are synonymous, not all independent sequences encode variant proteins. Vif variants with gross defects (e.g., premature stop codons) as well as those that were found to be inactive in the functional assay are designated “defective Vif.” The overall frequency of inactive Vif proteins is expressed as a percentage relative to the number of independent sequences.

Simple inspection of sequences for obviously inactivating mutations likely underestimates the frequency of inactive *vif* alleles, and would not identify weakly active Vif proteins. Therefore, we employed a functional test to measure Vif activity. We generated VSV-G pseudotyped HIV-1 vector particles in the presence of APOBEC3G or APOBEC3F and patient/isolate-derived Vif proteins and measured their infectivity using a reporter cell-line (Figure 2). Under these conditions, HIV-1 infectivity was reduced more than 100-fold by APOBEC3G and restored by HIV-1 (NL4–3) Vif to 35–45% of the level observed in the absence of APOBEC3G (Figure 2A). APOBEC3F was a less potent inhibitor of HIV-1 and reduced infectivity by 15- to 20-fold, as has been recently reported [11]. Additionally, APOBEC3F was more resistant to neutralization by NL4–3 Vif, which restored infectivity to about 15% to 25% of its uninhibited level, even at the maximum level of Vif expression tested. The linear range of the assay with respect to the amount of transfected Vif expression plasmid was determined (Figure 2A), and levels of Vif expression within this range were used in subsequent experiments.

We next selected a panel of 40 representative and apparently intact Vif proteins from the 79 independent clones and measured their ability to neutralize APOBEC3G antiviral activity. The majority of patient- and isolate-derived Vif proteins exhibited similar activity to that of NL4–3 Vif (Figure 2B). However, each set of patient-derived and isolate-derived Vif proteins contained at least one variant that was either inactive or only weakly active (Figure 2B). In fact, nine of the 40 apparently intact Vif proteins were non-functional despite the fact that they were well expressed. One additional amino-terminally truncated Vif variant (V4–8) that contained a stop at codon 11 but was expressed via an alternative translation initiation site at codon 16 was found to be inactive. Five additional *vif* variants (P2, P3) that contained premature stop codons were assumed to be inactive. Therefore, of the 79 sampled *vif* alleles, comprising 61 distinct proteins, at least 15 were unable or poorly able to counteract APOBEC3G antiviral activity (Figure 2C). Additionally, even among functional Vif variants, there was significant variation in the extent to which they restored HIV-1 infectivity that was not explained by variation in expression level (Figure 2B, and data not shown).

### Mutations Conferring Loss of APOBEC3G- and/or APOBEC3F-Neutralizing Activity

**Figure 3:**
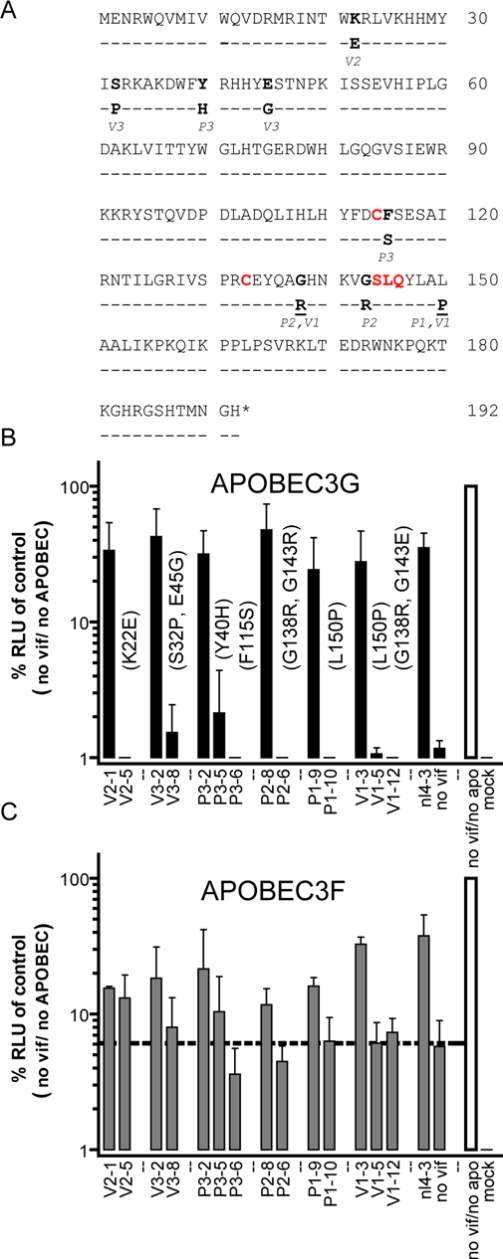
Closely Related Vif Proteins Display Distinct Functional Properties (A) Summary of the residues implicated as causing Vif defects by comparison of functional and non-functional Vif variants. Amino acid substitutions that occurred exclusively in non-functional Vif proteins are depicted relative to the NL4–3 Vif sequence. Changes identified by * are caused by G-to-A mutations. (B, C) Function of closely related *vif* alleles assessed by quantitation of the infectivity of particles produced in the presence of APOBEC3G (B) or APOBEC3F (C). Amino acid substitutions in the non-functional partner of the “matched” functional Vif variant are given in parentheses in (B). The dotted line in (C) indicates the level of infectivity observed for the vector generated in the presence of APOBEC3F and in the absence of Vif. The data represent the average infectivity values of at least three independent experiments, with the error bars showing the standard deviations.

To determine the amino acid substitutions responsible for functional inactivation of apparently intact Vif proteins, we identified the closest functional relative of each non-functional variant. In most cases, we could identify functional and non-functional Vif proteins that differed by only one or two amino acids (Figure S1). Of note, two mutations (G138R and L150P) that were found only in non-functional variants arose independently in unrelated Vif proteins (from P1, P2, and/or V1, Figure 3A). The only non-functional Vif variant for which no very closely related functional counterpart was identified was from patient DNA (P2–7) which differed at four positions from its closest functional relative (W11R, K63E, G75R, G185R) and was not analyzed further.

We tested the panel of “matched” functional and non-functional Vif variants for their relative ability to neutralize APOBEC3G and APOBEC3F. Like NL4–3 Vif, those that neutralized APOBEC3G also neutralized APOBEC3F, albeit less efficiently (Figure 3B and 3C). Generally, the matched variants that were inactive or weakly active against APOBEC3G also failed to neutralize APOBEC3F. However, two Vif variants (V2–5, P3–5) that contained mutations K22E and Y40H, respectively, were unusual in that they did not neutralize APOBEC3G but retained partial activity against APOBEC3F (Figure 3B and 3C).

To confirm these findings and to unambiguously identify the mutations responsible for loss of function, we constructed a panel of NL4–3 Vif mutants containing naturally occurring mutations that were associated with loss of APOBEC3G neutralization. The W11R mutant was added to this panel, as this was the only substitution in the non-functional P2–7 variant (which contained three additional substitutions, see above) that occurred at a position that is invariant in the HIV-1 sequence database.

**Figure 4:**
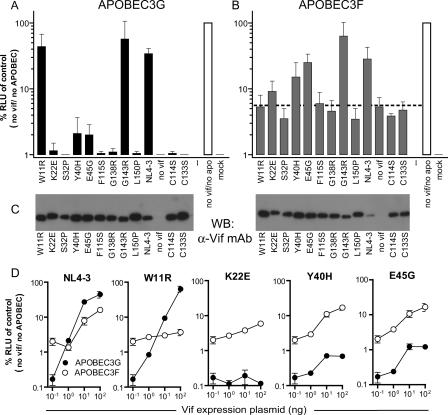
Effect of Naturally Occurring Single Amino Acid Substitutions on NL4–3 Vif APOBEC3G and APOBEC3F Neutralization Activity The HIV-1 vector infectivity generated in the presence of APOBEC3G (A) and APOBEC3F (B) and a fixed dose of NL4–3 Vif mutant was measured. The dotted line in (B) indicates the level of infectivity observed for the vector generated in the presence of APOBEC3F and in the absence of Vif. The data represent the average infectivity as determined by at least three independent experiments, with the error bars showing the standard deviations. (C) Protein expression levels of the NL4–3 Vif mutants were determined by Western blotting of transfected 293T cell lysates. (D) Infectivity of HIV-1 vector generated in the presence of APOBEC3G (filled symbols) and APOBEC3F (open symbols) and varying levels of selected Vif mutants.

In the presence of APOBEC3G, NL4–3 Vif harboring the mutations K22E, S32P, Y40H, E45G, F115S, G138R, or L150P failed to restore HIV-1 infectivity (Figure 4A). Conversely, NL4–3 Vif containing the mutations W11R or G143R displayed activity close to that of unmutated NL4–3 Vif. Most of the mutants exhibited similar loss or retention of activity against APOBEC3F as against APOBEC3G (compare Figures 4A and 4B). Western blotting of transfect cell lysates revealed that expression levels of the Vif mutants that fail to rescue particle infectivity were comparable or superior to the expression level observed for NL4–3 Vif (Figure 4C). Interestingly, mutants W11R, K22E, Y40H, and E45G displayed a selective propensity to neutralize one of the two APOBEC3 proteins. These mutants were analyzed in more detail by measuring viral infectivity in the presence of APOBEC3G or APOBEC3F and varying levels of Vif (Figure 4D). This analysis revealed that the K22E mutant retained partial activity against APOBEC3F but was inactive against APOBEC3G. The Y40H and E45G Vif mutants neutralized APOBEC3F activity almost as efficiently as wild-type NL4–3 Vif, but displayed only weak activity against APOBEC3G. Conversely, the W11R mutant exhibited the precisely opposite phenotype and neutralized APOBEc3G but not APOBEC3F. Thus, naturally occurring mutations can result in Vif variants that neutralize one but not the other cytidine deaminase.

### In Vivo Sequence Diversity Consistent with Selective Exposure to APOBEC3G or APOBEC3F

**Figure 5:**
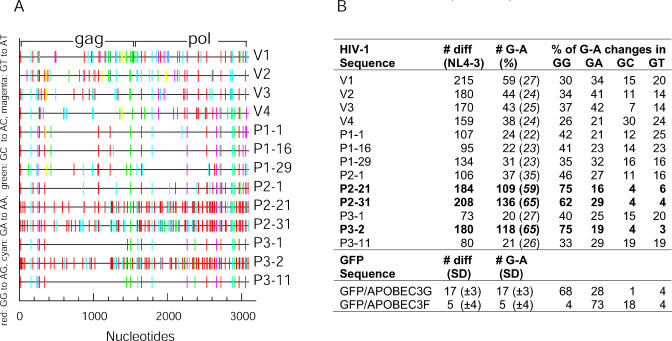
Analysis of *Gag-pol* Sequences Derived from LTNP and Viral Isolates (A) Graphic representation of the G-to-A changes (compared to HIV-1 NL4–3) present in *Gag-pol*. Analysis was performed using the HYPERMUT program [48]. (B) Quantitative summary of the observed changes. “# diff” is the number of positions at which the patient sequence differs from NL4–3. “# G-A (%)” represents the absolute number and percentage of all substitutions that are G-to-A changes. The dinucleotide context (GG, GA, GC, GT) reflects the two contiguous bases, with G-to-A mutations occurring in the first position. The numbers of differences (“# diff”) for the APOBEC3G- and APOBEC3F-induced changes in the in vitro assay are given as an average (± standard deviation) of those occurring in 10 to 15 clones of hrGFP (450 nucleotides).

In principle, mutations that selectively affect the ability of Vif to neutralize APOBEC3G and/or APOBEC3F could profoundly affect HIV-1 sequence evolution. Because we found defective Vif variants in patients who also contained G-to-A hypermutated viral DNA (P2 and P3) [31,33], we next obtained a more extensive sample of *Gag-Pol* sequences from patients and viral isolates harboring defective Vif alleles (a total of 25 independent *Gag-Pol* sequences of which 13 representative sequences are depicted in Figure 5).

In general, the *Gag-Pol* sequences from the LTNPs contained about 3-fold fewer changes relative to an NL4–3 HIV-1 reference sequence than did the viral isolate sequences. This is perhaps due to the fact that the three LTNPs became infected more than 20 y ago, quite early in the North-American HIV-1 epidemic and around the time the viral isolate NY5, which comprises the 5′ portion of the reference sequence NL4–3 [37], was obtained. Conversely, the viral isolates were derived from patients infected in the late 1990s. Nonetheless, the fraction of changes relative to the reference sequence that were G-to-A mutations was quite similar for patient and viral isolate sequences (about 25% of all changes) and higher than would be expected if nucleotide substitutions were random (Figure 5B). This phenotype was exaggerated in several P2- and P3-derived sequences that were clearly hypermutated. In some cases, examples of which are shown in Figure 5A and 5B, about 65–70% of all changes relative to the reference sequence were G-to-A changes. In P1, no obviously hypermutated viral sequences were detected, while in P2 and P3, viral DNA was often hypermutated and defective. Indeed, hypermutated Vif sequences were previously detected in P3 (see Figure 1).

In principle, G-to-A mutations can occur in one of four different dinucleotide contexts. We sequenced hrGFP-containing viral DNA generated following infection with HIV-1 vectors assembled in the presence of APOBEC3G or APOBEC3F. Consistent with recent reports [9,11], APOBEC3F primarily induced GA-to-AA changes and very few GG-to-AG changes in hrGFP. Conversely, APOBEC3G caused twice as many GG-to-AG as GA-to-AA changes (Figure 5B). In the sequences from all the isolate-derived DNA as well as in some patient-derived *Gag-Pol* clones (e.g., P1–29, P3–11; Figure 5B), GA-to-AA changes were more prevalent than GG-to-AG changes, relative to the reference sequence, suggesting that HIV-1 is more often mutated by APOBEC3F than by APOBEC3G. In contrast, in the extensively hypermutated *Gag-Pol* clones (e.g., P2–21, P2–31, P3–2, Figure 5A and 5B) there was an approximately 2:1 ratio of GG-to-AG versus AG-to-AA substitutions, which closely recapitulated the result of APOBEC3G- (and not APOBEC3F-) induced hrGFP mutagenesis.

**Figure 6:**
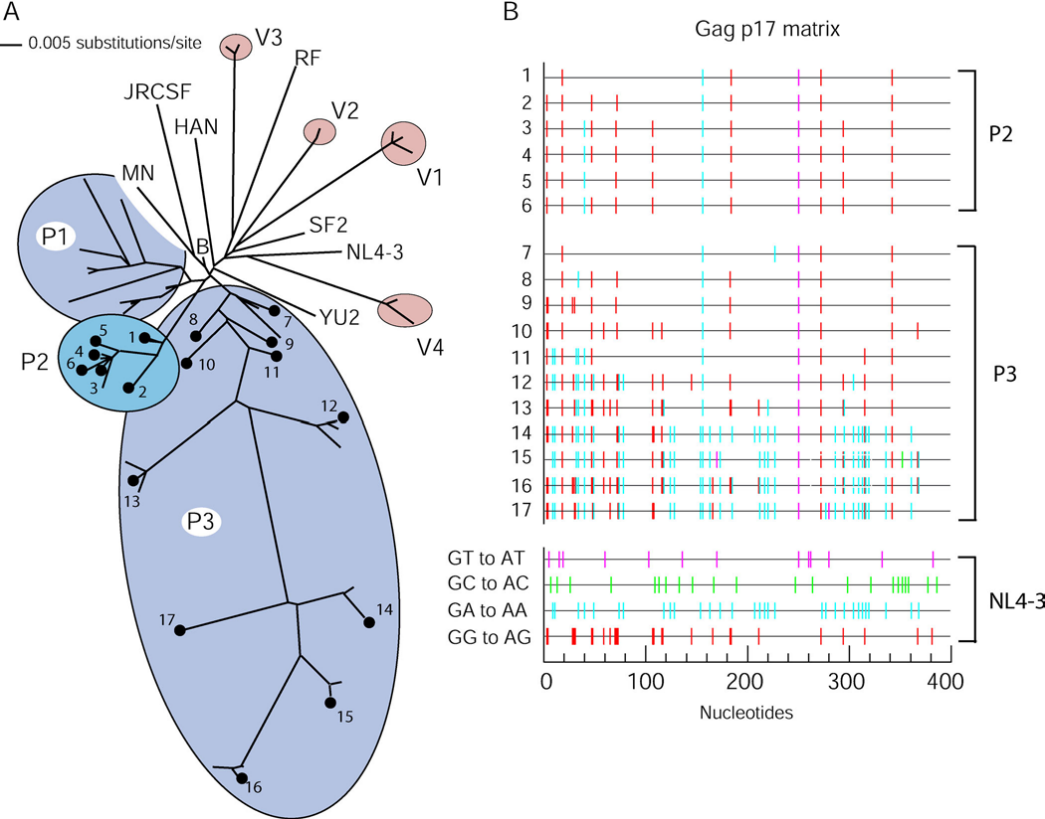
Phylogenetic Relationships and Hypermutation in p17MA Sequences (A) Neighbor joining tree representing the phylogenetic relationships among 70 independent p17MA sequences derived from patients (P1, P2, P3) and viral isolates (V1, V2, V3, V4). Additional subtype B reference sequences were also included. (B) Graphic representation of the G-to-A changes (compared to HIV-1 NL4–3) present in p17MA sequences of P2 and P3. Analysis was performed using the HYPERMUT program [48]. All possible G-to-A substitutions in the context of the reference sequence (NL4–3) are shown with the dinucleotide context color coded (bottom four sequences).

Because the p17MA region of *Gag* provided a reasonable representation of mutations present in *Gag-Pol,* and because p17MA sequences present in P3 had previously been extensively sampled [31], we restricted a more detailed phylogenetic analysis to this region. Thus, the larger dataset comprised a total of 70 p17MA sequences, and the phylogenetic analysis is depicted in Figure 6A. Notably, the p17MA sequences obtained from a single individual harboring numerous hypermutated proviruses (P3) were exceptionally heterogeneous and exhibited greater diversity than that observed among a selection of subtype B reference sequences. While very diverse hypermutated sequences co-existed in a patient and sometimes within a single sample (Figure 6B), sampling bias due to the very low number of proviruses present in these LTNPs makes it difficult to derive conclusions about temporal variation in the proportion of hypermutated to “normal” sequences. Nonetheless, it was clear that in some individuals at some time-points, hypermutated sequences predominated. Notably, individual p17MA clones contained distinct patterns of G-to-A substitutions that reflect the generation of multiple, independently hypermutated proviruses within a single individual. Moreover, some clones almost exclusively harbored GG-to-AG changes relative to the reference sequence (e.g., P2–3, P2–5, P3–8, P3–9, Figure 6B), while in others GA-to-AA changes predominated (e.g., P3–13, P3–14, Figure 6B). In other clones, GG-to-AG and GA-to-AA substitutions occurred in variable proportions. This bias recapitulated the footprints expected to result from mutation induced either by APOBEC3G, APOBEC3F, or a mixture of both and is likely, therefore, the result of variation in the ability of Vif, within an individual, to neutralize these cytidine deaminases.

## Discussion

Host cells use DNA-editing enzymes such as APOBEC3G and APOBEC3F to inhibit the replication of exogenous and endogenous retroviruses [38,39]. In turn, lentivirus Vif proteins counter the antiretroviral activity of these cytidine deaminases [5–12] by inducing their degradation.

While defective *vif* alleles should render the majority of HIV-1 progeny virions noninfectious, establishment of proviruses that have APOBEC3G- and/or APOBEC3F-induced mutations should endow the proviral population with additional diversity beyond that resulting from error-prone reverse transcription [1,3]. Here, we show that defective *vif* alleles are easily found, even in a relatively small sample of viral isolates and uncultured viral DNA from infected individuals. Because active and defective Vif variants were detected in similar proportions (about 19%) in LTNP DNA and viral isolates, it seems probable that Vif is sporadically inactivated at some frequency by reverse transcriptase errors or cytidine deamination in all HIV-1-infected individuals. We also found multiple, independent hypermutated proviruses in two of the three LTNPs studied. It is likely that the relative ease of detection of extensively hypermutated proviruses is an unusual property of LTNPs [26] and hypermutated proviruses are only rarely detected in other situations because they are obscured by superimposed replication competent virus. Put another way, the ease of detection of hypermutated HIV-1 is likely a consequence, rather than a cause, of LTNP status.

A recent report provides independent evidence for the occurrence of sporadic Vif inactivation in vivo: indeed, more than 9% of proviral sequences derived from resting CD4+ cells of HAART-treated patients with plasma viremia below the level of detection were found to be hypermutated [40].

Site-directed mutagenesis studies revealed a number of residues and domains located throughout Vif that are essential for infectivity and viral replication in non-permissive cells [41–44]. We identified here several examples of naturally occurring mutations in Vif that induce selective defects in APOBEC3G- or APOBEC3F-neutralizing activity. Interestingly, these occurred toward the amino-terminus of Vif, while those causing general defects occurred either near the amino-terminus or close to the functionally important SLQ motif [41,42,45]. These findings suggest the possibility that the Vif amino-terminal domain contains determinants that confer specific binding to APOBEC3G or APOBEC3F. Such mutations should cause nascent viral DNA to be exposed either to APOBEC3G or APOBEC3F or both, with the induction of very different patterns of G-to-A changes in the viral quasi-species. Due to the nature of sampling proviral DNA in a patient, it is practically impossible to link a particular Vif point mutant to a subsequently generated hypermutated provirus. Nonetheless, even within a tiny fraction of the total burden of, e.g., P3′s HIV-1 sequences, Vif variants that selectively failed to neutralize APOBEC3G as well as p17MA sequences bearing the footprint of selective mutation by either cytidine deaminase were rather easily detected (Figure 6). Selective and sporadic loss of cytidine deaminase neutralization can result in massive sequence variation. Indeed, the extent of p17MA sequence diversity within one of the individuals studied herein (P3) surpassed the variation observed among a selection of subtype B reference sequences and contemporary isolates. While the bulk of hypermutated proviruses are replication defective, they could, in principle, provide a genetic “resource,” and selected fragments could easily be recombined into the circulating viral population as cells harboring them become super-infected. Moreover, while intermittent inactivation of Vif has the measurable consequence of deposition of hypermutated proviruses, this likely represents an extreme manifestation of the loss-of-function phenotype. It is quite likely that more subtle (and less easily measurable) variation in Vif's anti-APOBEC3G and anti-APOBEC3F activities occurs at greater frequency than the roughly 20% inactivation documented herein. It is also interesting that some of the mutations that affected Vif function were themselves the result of G-to-A changes. Thus, “feedback” loops of Vif mutation resulting in more or less mutation of Vif and other viral genes could result from variation in Vif function.

The high adenosine content of lentiviral genomes [22] and the sporadic detection of hypermutated genomes in vivo [23–26] suggest that cytidine deamination provides an important source of viral diversification. Thus, an activity that likely evolved as a host defense against viruses might have been usurped and may facilitate HIV-1 escape from immunological and pharmacological inhibition. In addition, an appreciation of the respective contributions of viral and host-mediated mutagenesis to viral diversity may be important for determining the potential efficacy and the potential risks associated with the use of antiretroviral strategies that target vif:APOBEC3G/3F interactions.

## Materials and Methods

### Patient-derived and NL4–3 mutant *vif* alleles.

DNA was extracted from either patient's PBMCs (P1, P2, and P3), or from PBMCs used for viral propagation (V1, V2, V3, V4) using DNeasy DNA isolation kit (Qiagen, Valencia, California, United States). Samples were obtained in 1995 and 2000 for P1, in 2000 and 2001 for P2, and 2003 for P3. Because clinical materials from the mid-1990s were not available for P3, we reconstructed the two P3′s vif alleles that were previously described and contained amino acid substitutions relative to a P3 sequence from 2003 (Y40H and F115S [28]). Full-length *vif* genes were amplified by nested PCR using high-fidelity polymerase and cloned into the expression vector pCRV1, as previously described [46]. In order to minimize sampling bias due to the very low proviral load in some LTNP samples (e.g., less than five copies/10^6^ PBMCs [30]), multiple parallel PCR reactions were done. Cloned Vif variants were sequenced in both directions and aligned using the DNAStar (Madison, Wisconsin, United States) Version 4.03 software package. Phylogenetic relationships were assessed by the Neighbor Joining method (PAUP software). Site-directed mutations were introduced into NL4–3 Vif using mutagenic oligonucleotides and recombinant PCR resulting in NL4–3 Vif mutants encoding W11R, K22E, S32P, Y40H, E45G, F115S, G138R, G143R, and L150P as well as the previously described mutants C114S and C133S [47]. Wild-type and mutant NL4–3 Vif proteins as well as the patient- and primary-isolate-derived Vif variants were expressed using pCRV1.

### *Gag-Pol* and p17MA sequence analysis.

A region spanning the Gag, protease (PR), and half of the reverse transcriptase gene (RT; total 3,094 nucleotides) was amplified from all three LTNPs and the four viral isolates using nested PCR and the same DNA samples used as a source of *vif* variants. PCR fragments were cloned into TOPO XL vector (Invitrogen, Carlsbad, California, United States) and sequenced bidirectionally. To ensure appropriate sampling in the setting of low proviral load, we performed multiple PCR reactions in parallel and cloned the fragments from five to seven PCR reactions separately. Phylogenetic relationships were assessed by the Neighbor Joining method (PAUP software). Analysis of the G-to-A substitutions was performed using the HYPERMUT program [48].

### Assay for Vif function.

HIV-1 vector particles were generated by transfecting 293T cells with plasmids expressing HIV-1 gag-pol (pCRV1/Gag-pol)[49], a packagable HIV-1 RNA genome that encodes only Tat, Rev, Vpu, and GFP (pV1/hrGFP) and the G protein from vesicular stomatitis virus (pHCMV VSV-G) in a 5:5:1 ratio. To measure Vif function, cells were co-transfected with this plasmid mixture and additional plasmids expressing amino-terminally HA-tagged APOBEC3G or APOBEC3F and a pCRV1/Vif variant. Cells were transfected in 24 well plates using LipoFectamine Plus reagent (Invitrogen) and the supernatant harvested 48 h later and filtered. Infectivity was measured in duplicate assays using TZM-bl cells, which carry an HIV-1 Tat responsive β-galactosidase indicator gene under the transcriptional control of the HIV-1 promoter. Infection was done in the presence of 8μg/ml Polybrene, and β-galactosidase activity was quantified 48 h later using chemiluminescent substrate as previously described [50].

### Measurement of APOBEC3G- and APOBEC3F-driven hrGFP mutagenesis.

HIV-1 vector particles were generated by transfection of 293T cells, as for the analysis of Vif function with two differences: First, APOBEC3G or APOBEC3F but no Vif expression plasmid was included in the transfection mixture. Second, to minimize the possibility that transfected DNA rather than de novo synthesized viral DNA would be amplified and sequenced, the pV1/hrGFP plasmid vector was omitted from the transfection mixture and a 293T-derived cell line carrying an integrated V1/hrGFP vector genome was used to generate vector particles. Vector particles generated by this method were used to infect MT4 cells and DNA was extracted 8 to 10 h later using a DNeasy DNA isolation kit. hrGFP sequences were amplified by PCR, cloned into TOPO vector, and sequenced bidirectionally. Sequences (450-nucleotide fragment) from 10 to 15 clones were aligned using the DNAStar software package.

## Supporting Information

Figure S1 The Sequences of Functional and Non-Functional Vif Alleles (Underlined) Were Compared in Order to Identify Positions Relevant for Inactivation of Vif (24 KB PDF)

Table S1 Summary of Clinical and Virological Characteristics of the Four Patients from Whom Virus Was Cultivated As Well As from the Three LTNPs Studied (16 KB PDF)

### Accession Numbers

The GenBank (http://www.ncbi.nlm.nih.gov/Genbank) accession numbers for the *vif* and *gag-pol* sequences are DQ097739–DQ097768.

## Abbreviations

LTNP: long-term non-progressor
PBMC: peripheral blood mononuclear cell
