# Supplementary material for: Natural Variation in Vif: Differential Impact on APOBEC3G/3F and a Potential Role in HIV-1 Diversification
Source: PLoS Pathog. 2005 Jul 22;1(1):e6. doi: 10.1371/journal.ppat.0010006 (PMC1238741; doi:10.1371/journal.ppat.0010006)
Supplement: Figure S1 — (24 KB PDF) [file ppat.0010006.sg001.pdf]

Supplementary Figure 1 online
(Simon et al.)

```
                           11          22         32      40     45
NL4-3           MENRWQVMIV WQVDRMRINT WKRLVKHHMY ISRKAKDWFY RHHYESTNPK ISSEVHIPLG DAKLVITTYW GLHTGERDWH LGQGVSIEWR KKRYSTQVDP
P1-9            ---------- --------R- --S------- ------G--- -------H-R ---------- ---------- ---------- ---------- ----------
P1-10           ---------- --------R- --S------- ------G--- -------H-R ---------- ---------- ---------- ---------- ----------
P2-8            ---------- --------RA --S------- R-G---G--- -------H-R ---------- ---------- ---------- ---------- ----N-----
P2-6            ---------- --------RA --S------- R-G---G--- -------H-R ---------- ---------- ---------- ---------- ----N-----
P2-7            ---------- R-------RA --S------- R-G---G--- -------H-R ---------- --E------- ----R----- ---------- ----N-----
P3-2            ---------- --------R- --S------- --G---G--- -------H-R ---------- --R------- ---------- ---------- -R--------
P3-5            ---------- --------R- --S------- --G---G--H -------H-R ---------- --R------- ---------- ---------- -R--------
P3-6            ---------- --------R- --S------- --G---G--- -------H-R ---------- --R------- ---------- ---------- -R--------
V1-3            ---------- --------RA --S------- --G--RK--- -------H-R ---------- E----V---- ---------- ---------- QR--------
V1-5            ---------- --------RA --S------- A-G--RK--- -------H-R ---------- E----V---- ---------- ---------- QR--------
V1-12           ---------- --------RA --S------- --G--RK--- -------H-R ---------- E----V---- ---------- ---------- QR--------
V2-1            --------V- --------K- --S------H ---R-SG--- ------RH-- ---------- --R--V---- ---A------ ---------- QR--------
V2-5            --------V- --------K- -ES------H ---R-SG--- ------RH-- ---------- --R--V---- ---A------ ---------- QR--------
V3-2            ---------- --------R- --S------H --G---G-V- ------I--R ---------- ---------- ---------- ---------- -G--------
V3-8            ---------- --------R- --S------H -PG---G-V- ----G-I--R ---------- ---------- ---------- ---------- -G--------
V4-1            ---------- --------R- -NS---Y--- V-K--SG--- -----NPH-R -N-------- ------A--- ---------- ---------- -R--R---T-
V4-8            ---------- *-------R- -NS---Y--- V-K--SG--- -----NPH-R -N-------- ------A--- ---------- ---------- -R--R---T-    100

                               115                      138   143    150
NL4-3           DLADQLIHLH YFDCFSESAI RNTILGRIVS PRCEYQAGHN KVGSLQYLAL AALIKPKQIK PPLPSVRKLT EDRWNKPQKT KGHRGSHTMN GH*
P1-9            N--------Y ---------- --A-V-H--- -S-------- =--------------T--K-- ------T--- ---------- ---------- ---
P1-10           N--------Y ---------- --A-V-H--- -S-------- ---------P ----T--K-- ------T--- ---------- ---------- ---
P2-8            N--------Y -------T-- --A------- ---------- ---------- ----T--K-- ------T--- ---------- ---------- ---
P2-6            N--------Y -------T-- --A------- -------R-- --R------- ----T--K-- ------T--- ---------- ---------- ---
P2-7            N--------Y -------T-- --A------- ---------- ---------- ----T--K-- ------T--- ---------- ----R----- ---
P3-2            ---------Y ---------- --A---H--- -S-------- ---------- ----T--K-- ------T--- ---------- ---------- ---
P3-5            ---------Y ---------- --A---H--- -S-------- ---------- ----T--K-- ------T--- ---------- ---------- ---
P3-6            ---------Y ----S----- --A---H--- -S-------- ---------- ----T--K-- ------T--- ---------- ---------- ---
V1-3            ---------- ---------- -KAL--YR-T ---------- ---------- T---A--K-- ---------- --------R- ---------- ---
V1-5            ---------- ---------- -KAL--YR-T ---------- ---------P T---A--K-- ---------- --------R- ---------- ---
V1-12           ---------- ---------- -KAL--YR-T -------R-- --E------- T---A--K-- ---------- --------R- ---------- ---
V2-1            G-------QY ---------- --A---H--- ---------- ---------- T-----RKV- ---------- ---------- ---------- ---
V2-5            G-------QY ---------- --A---H--- ---------- ---------- T-----RKV- ---------- ---------- ---------- ---
V3-2            G-T-R----Y -------T-- --A---H--- -S-------- ---------- T--R---K-- ------A--- ---------- ---------- ---
V3-8            G-T-R----Y -------T-- --A---H--- -S-------- ---------- T--R---K-- ------A--- ---------- ---------- ---
V4-1            ---------Y ------D--- -KA---HR-- ------T--- ---------- ----T--KR- ------A--- ---------- ---------- ---
V4-8            ---------Y ------D--- -KA---HR-- ------T--- ---------- ----T--RR- ------A--- ---------- -----N---- ---          192
```

Supplemental Figure 1: The sequences of functional and non-functional vif alleles (underlined) were compared in order to identify positions relevant for inactivation of Vif. The residues in which the alleles differ are depicted in bold. In some instances the closest related functional allele differed in more than one residue from its non-functional counterpart. * at position 11 of V4-8 depicts a premature stop codon.
