# Supplementary material for: Natural Variation in Vif: Differential Impact on APOBEC3G/3F and a Potential Role in HIV-1 Diversification
Source: PLoS Pathog. 2005 Jul 22;1(1):e6. doi: 10.1371/journal.ppat.0010006 (PMC1238741; doi:10.1371/journal.ppat.0010006)
Supplement: Table S1 — (16 KB PDF) [file ppat.0010006.st001.pdf]

Supplementary Table 1 online
(Simon et al.)

**Patient**	**Risk**	**Duration of infection**	**CD4 T lymphocytes** (cells/mm^3)	**HIV-1 RNA** (copies/ml)
V1	MSM	<2 months	263	>1 500 000
V2	n.k.	chronic	<500	>1 000 000
V3	MSM	<1 month	475	>1 000 000
V4	MSM	<1 month	423	>500 000
P1	MSM	>22 years	560-740	<50
P2	IVDU	>21 years	800-1000	<50
P3	IVDU	>21 years	500-700	<50

Supplementary Table 1: Summary of clinical and virological characteristics of the four patients from whom virus was cultivated as well as from the three long term non-progressors (LTNP) studied.
n.k.: not known, IVDU: intra-venous drug use; MSM: men that have sex with men
